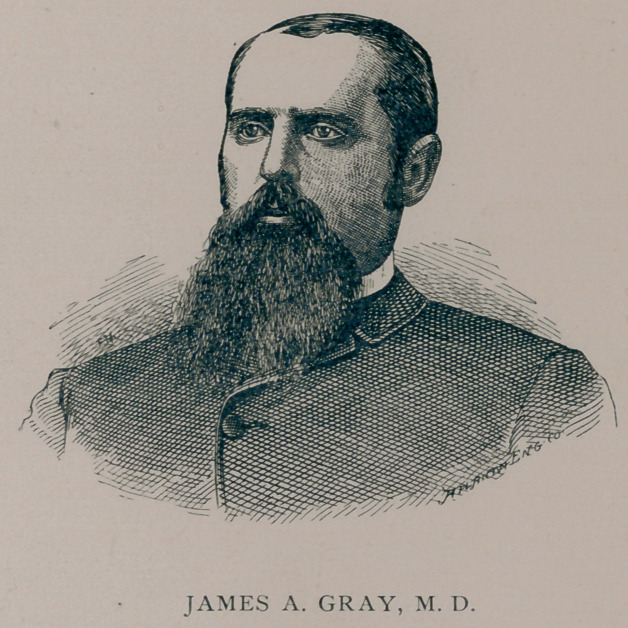# In Memoriam—Dr. James A. Gray

**Published:** 1887-10

**Authors:** 


### Editorial.

### IN MEMORIAM.

Dr. James A. Gray was born in Monroe county, Georgia, thirty-seven years ago, and died at his residence in Atlanta on the 27th of September.

At the age of twenty-seven he began the study of medicine, and graduated with first honor at the Atlanta Medical College in 1879. He entered at once into practice in Atlanta, and with astonishing rapidity made for himself a place in the front ranks of his profession.

In 1881 he was made Proctor of the Atlanta Medical College and Lecturer on Venereal Diseases. At the time of his death, he was still occupying these positions, and was also serving his second term as Secretary of the Medical Association of Georgia. He was surgeon of the Atlanta Rifles, a member of the Atlanta Society of Medicine, and managing editor of The Atlanta Medical and Surgical Journal.

During the session of 1880–81, he was Demonstrator of Anatomy in the Southern Medical College.

He died of typhoid fever after a short illness of about three weeks.

Death has closed prematurely a career characterized from first to last by phenomenal success.

Dr. Gray was a man of remarkable ability. He crowded into eight short years of professional work the achievements of a long

JAMES A. GRAY, M. D.

life, and at an age when most men are still sowing the seeds of success, he was reaping a rich harvest. The key-note to his success was energy, ceaseless and tireless. Quick to grasp a situation, and quicker still to act, he succeeded in whatever he undertook. Every blow which he struck went straight to the mark with telling effect. His policy in every undertaking was bold, open and aggressive. Such qualities made him an invaluable officer of the Atlanta Medical College. As Proctor, he did an inconceivable amount of good to this school by bringing into the management of its affairs his shrewd business sagacity, and ever since his connection with it, it has enjoyed an unprecedented prosperity. As Lecturer on Venereal Diseases, it is sufficient to say that he gave universal satisfaction to his students.

In 1884 Drs. Westmoreland, Miller and Gray took editorial control of the ATLANTA MEDICAL AND SURGICAL JOURNAL, a medical monthly which was founded in 1855. Dr Gray at once devoted himself, heart and soul, to the conduct of this JOURNAL, and in this, as in every other undertaking, his efforts were speedily crowned with success. At present THE JOURNAL has the largest circulation and the best advertising patronage of any medical periodical in the South. Its prosperity is a lasting monument to Dr. Gray's wonderful ability.

His work as Secretary of the Medical Association of Georgia is well known to the whole profession of the State. He received the unqualified endorsement of the Association at its last annual meeting by being re-elected to the position he had filled so creditably.

He was a most valued member of the Atlanta Society of Medicine, although his arduous duties did not allow him to attend its meetings with regularity ; whenever he did take part in its discussions, his remarks were always characterized by that sound, practical sense for which he was so noted. As a physician, he was greatly beloved by his patients ; his strong character, emphatic manner of speech and sound judgment gave them boundless confidence in him. As a diagnostician, he was very accurate, his knowledge of disease being almost intuitive. Although he achieved a substantial reputation as physician, as lecturer and as editor, it was not in these respects that he shone most. The qualities of his brain were subordinate to those of his great, generous heart. His tender devotion to his wife was the most beautiful phase of his character, and beyond doubt, he drew his inspiration from her loving encouragement. As a friend, he was generous and true, always ready to work for those he loved without one thought of himself ; indeed, one of the last acts of his life illustrated his unselfish devotion as a friend ; just one week before he died, he rose from his bed of sickness and successfully pushed the interests of one of his best friends.

A good man has been taken—struck down in the zenith of his glory, leaving desolate the hearts of two aged parents and a loving wife.

His body was laid to rest in Westview Cemetery, the burial services being conducted by the Masonic fraternity, of which he was a distinguished member. The medical profession attended the funeral in a body.

RESOLUTIONS OF RESPECT.

At a meeting of the Atlanta Society of Medicine, held September 27, 1887, the committee charged with the duty of "preparing some suitable memorial of our personal friend and professional brother, Dr. James A. Gray," respectfully submitted the following resolutions:

*Resolved*, That it is the sense of the Atlanta Society of Medicine that in the early and unexpected death of our dear brother, the medical profession of this city and of the State at large has sustained a serious loss, not less by the purity of his life and the amiability of his character than by his acknowledged ability. As a professor, editor and practitioner, he had greatly impressed his professional brethren and the general community. Cut off in the midst of his days, he has left a life-record not only untarnished, but even resplendent with the noble deeds prompted by noble and unselfish aims.

*Resolved*, That we tender to his devoted wife and other kindred our sincere condolence in their sad bereavement.

*Resolved*, That a blank page of our Society minutes, inscribed with his name, be dedicated to his honored memory.

*Resolved*, That we attend in a body his funeral, and that this tribute be published in *The Atlanta Constitution* and *Evening Journal.*

(Signed) W. S. ELKINS, M. D.,
N. O. HARRIS, M. D.,
H. P. COOPER, M. D.,
W. F. WESTMORELAND, JR., M. D.,
H. F. SCOTT, M. D.,
Committee.

## Figures and Tables

**Figure f1:**